# Sustained Complete Response to Metronomic Chemotherapy in a Child with Refractory Atypical Teratoid Rhabdoid Tumor: A Case Report

**DOI:** 10.3389/fphar.2017.00792

**Published:** 2017-11-01

**Authors:** Mahe Berland, Laetitia Padovani, Angélique Rome, Grégoire Pech-Gourg, Dominique Figarella-Branger, Nicolas André

**Affiliations:** ^1^Department of Pediatric Hematology-Oncology, Assistance Publique Hôpitaux de Marseille, La Timone Hospital, Marseille, France; ^2^Department of Radiation Oncology and Pediatrics, Assistance Publique Hôpitaux de Marseille, La Timone Hospital, Marseille, France; ^3^Department of Neurosurgery, Assistance Publique Hôpitaux de Marseille, La Timone Hospital, Marseille, France; ^4^Department of Pathology, Assistance Publique Hôpitaux de Marseille, La Timone Hospital, Aix-Marseille University, Marseille, France; ^5^Centre de Recherche en Oncologie Biologique et en Oncopharmacologie, INSERM-UMR 79 911, Aix-Marseille University, Marseille, France; ^6^Metronomics Global Health Initiative, Marseille, France

**Keywords:** pharmaceutical preparations, metronomic chemotherapy, drug repurposing, brain tumor, ATRT, pediatric oncology

## Abstract

Atypical teratoid rhabdoid tumor (ATRT) is a rare and highly aggressive embryonal tumor of the central nervous system with a dismal prognosis and no definitive guidelines for treatment, especially at relapse or in case of refractory disease. Metronomic chemotherapy (MC) has emerged as a new treatment option in solid malignancies, with lower toxicity and is frequently combined with drug repositioning. We report a case of ATRT in an 8-year-old boy who progressed during multimodal therapy including surgical resection, chemotherapy and radiotherapy. He was treated with MC involving continuous oral celecoxib with alternating metronomic etoposide and cyclophosphamide, in combination with biweekly bevacizumab and monthly intrathecal liposomal cytarabine. To date, he remains clinically and symptomatically disease-free with a follow-up of 10 months. The treatment was well-tolerated. Metronomics represent a possible alternative regimen for children with recurrent or progressive ATRT.

## Introduction

Atypical teratoid rhabdoid tumor (ATRT) is a rare and highly aggressive embryonal tumor of the central nervous system with a dismal prognosis (Dufour et al., 2012). Because the tumor is so rare and randomized controlled trials are so few, there is no consensus on optimal treatment for children with ATRT. Different multimodal strategies consisting of surgery, chemotherapy, and radiation therapy are currently being used in an attempt to improve outcomes (Ginn and Gajjar, 2012).

A recent review of the US Cancer Database from 2004 to 2012 shows that trimodality therapy seems to offers the best outcome (Fischer-Valuck et al., 2016). Another review of the literature points out high dose chemotherapy with stem cell rescue and radiotherapy as the two key components associated with improved outcome in children and adolescents with newly diagnosed ATRT (Schrey et al., 2016). For patients with localized disease who received trimodality therapy, the overall survival rate at 5 years approaches 50% (Fischer-Valuck et al., 2016). But the outcome remains poor for children with a metastatic disease, bringing down the 5-years overall survival rate to 30%, and for children younger than 3 years old who are less likely to receive radiation therapy in regard to potential long term neurocognitive sequelae.

Metronomic chemotherapy (MC) is an emerging alternative approach to conventional chemotherapy, relying on more frequent and low-dose drug administrations with minimal drug-free breaks. It is a multi-targeted therapy which targets tumor angiogenesis, increases the anticancer immune response and induces tumor dormancy (Pasquier et al., 2010; André et al., 2014). MC is frequently associated with drug repositioning to generate what has been defined as metronomics (André et al., 2013).

Herein, we report a case of refractory metastatic ATRT which was successfully put into sustained complete remission with metronomics.

## Case Presentation

The patient, an 8-year-old boy without significant past medical history, presented with seizures in October 2015. Initial magnetic resonance imaging (MRI) examination revealed a heterogeneously enhanced solid and cystic mass with hemorrhagic areas in the left frontal cortex. The solid part of the tumor showed isointense signal with heterogeneous enhancement on T1-weighted images and slightly hyperintensity on T2-weighted images. There was MRI evidence of tumor in the intracranial leptomeninges at diagnosis. On admission, a partial tumor resection was performed. Pathologic diagnosis of ATRT grade IV of the World Health Organization (WHO) was confirmed by immunohistochemistry demonstrating loss of nuclear expression of INI1. No malignant cell was found into the cerebro-spinal fluid (CSF).

Because of the aggressive nature of the tumor a second surgery for further resection was performed on admission day 20. Chemotherapy was then started. The patient received two courses of chemotherapy with vincristine (1.5 mg/m^2^ D1), cyclophosphamide (800 mg/m^2^ D1–D3), and doxorubicin (30 mg/m^2^ D1, D2) but he progressed rapidly. In December 2015, the patient presented with clinical neoplastic meningitis, confirmed by identification of circulating tumor cells in the CSF and leptomeningeal enhancement with no local evolution of the tumor on the brain and spinal MRI. Patient received a second line of chemotherapy consisting in cisplatin (20 mg/m^2^ D1–D5), etoposide (100 mg/m^2^ D1–D3), and ifosfamide (1.5 g/m^2^ D1–D5) while waiting for radiation therapy. Radiation therapy was started 2 weeks later with a craniospinal irradiation of 36 Gy and a focal boost of 54 Gy. The follow-up MRI a month after the end of radiotherapy was clear. Patient received a second course of chemotherapy consisting in cisplatin, etoposide, and ifosfamide. In April 2016, the patient relapsed with neoplastic meningitis and presented headaches and mild alteration of consciousness MC was started right away according to the MEMMAT-derived protocol (Pasquier et al., 2011; Peyrl et al., 2012) without thalidomide and intrathecal etoposide (**Table 1**).

**Table 1:** Metronomic chemotherapy regimen: dosing schedule.

Bevacizumab	10 mg/kg	Intravenous	Days 1, 15
Liposomal cytarabine	35 mg	Intrathecal	Day 1
Celecoxib	400 mg	Oral	Continuous
Cyclophosphamide	2.5 mg/kg	Oral	Alternating 21 day cycles
Etoposide	50 mg/m^2^		


**FIGURE 1:**
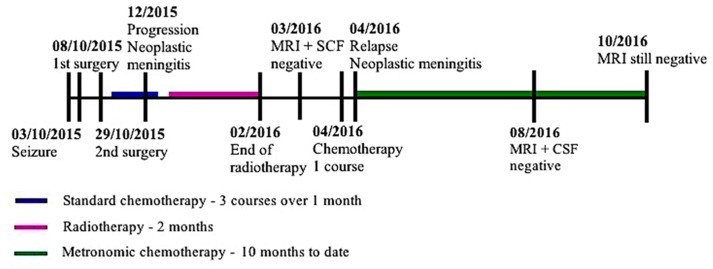
Timeline of diagnosis and treatment.

Symptoms rapidly subsided. MRI showed regression of the leptomeningeal enhancement a month after starting therapy and cleared up at month 4 (**Figure 1**). No circulating tumor cells could be found in the CSF any longer.

To date, the patient has received 10 months of MC. As for toxicity associated with the metronomic chemotherapy the patient exhibited moderate asthenia, 2 chemotherapy-induced reversible grade 4 neutropenia, type A influenza, and 2 reversible local reactions at the site of injection of intrathecal chemotherapy. No transfusion were necessary.

**FIGURE 2:**
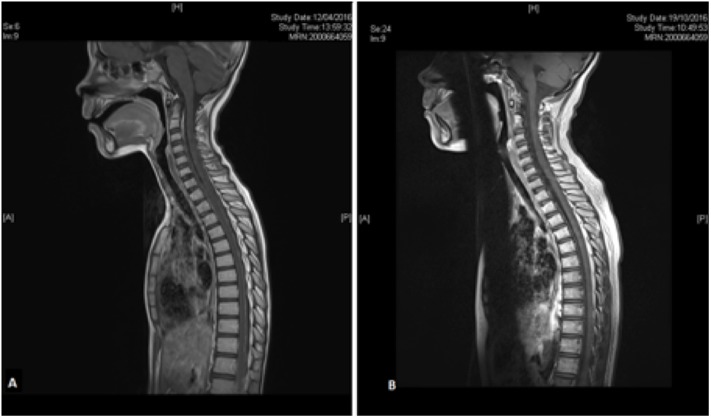
Spine MRI before and after 7 months of metronomic chemotherapy. MRI evidence of disseminated leptomeningeal tumor in pre-treatment T1-weighted gadolinium enhanced sagittal view **(A)** disappeared after 7 months of metronomic chemotherapy **(B)**.

He has had no complaints and has resumed normal activities, including going to school and participating in physical education. At last follow up, brain and spinal MRI is clear (**Figure 2**) and so is CSF examination. We plan to keep him on treatment for 12 months.

## Discussion

More clinical experience of MC in pediatric oncology has been gained over the past few years (Pasquier et al., 2010; André et al., 2014). Peyrl et al. (2012) reported the results of a multidrug combination regimen in 16 patients with recurrent embryonal brain tumors, including three ATRT. The regimen included continuous oral celecoxib, thalidomide, and fenofibrate with alternating metronomic etoposide and cyclophosphamide, in combination with biweekly bevacizumab, with or without intrathecal chemotherapy. All three patients with ATRT were alive at 42, 12, and 10 months after their last recurrence/progression; one showed initial partial response, one had stable disease and one had no evidence of disease after surgery.

Robison et al. (2014) presented data from a phase II 5-drug oral regimen in 101 patients with recurrent or progressive cancer, including two ATRT. Treatment consisted of MEMMAT without bevacizumab and intrathecal therapy. Clinical benefit was seen in patients with low-grade glioma and ependymoma, as well as in the miscellaneous stratum which included the two patients with ATRT. Specific results for these patients were not reported. Elsewhere, Gotti et al. (2015) have reported the response of a relapsing spinal ATRT to a metronomic combination of vinorelbine, cyclophosphamide, and celecoxib further confirming the potential of metronomics in this disease. Lastly, metronomics may also be useful when used as maintenance treatment (Malik et al., 2014) as reported by Choi et al. (2008) with high risk pediatric brain tumors including ATRT. Anyhow, while we do not know yet which kind of tumors a good candidate for a metronomic approach and in which setting (relapse, maintenance, radiation therapy sensitization), it is now admitted that different tumors may require different metronomic treatment (André et al., 2014).

Metronomic chemotherapy is generally well-tolerated in trials, with reports of predominantly mild toxicities. But careful patient selection is key; a recent study exploring the effect of metronomic thalidomide, etoposide, and celecoxib in children with various refractory or high risk tumor showed an improvement in quality of life measured by the Karnofsky-Lansky scores, although the majority of patients declared grades III–V adverse effects (Porkholm et al., 2016). The most common adverse effect observed is hematological toxicities, such as neutropenia that our patient exhibited. The absence of thalidomide in the regimen we used protected the patient from an increased risk of neurological toxicity. Besides, we decided to omit intrathecal etoposide since the patient, it would have required the insertion of an Omaya reservoir which was judged to be too invasive. Further investigations are warranted regarding potential long-term toxicities.

How the metronomics combination have led to achievement of a complete response in a refractory/relapsing disease can only be speculative and surely rely on several mechanisms. Indeed, MC has been reported to be able to inhibit angiogenesis as well as restore the anticancer properties of the immune system (Pasquier et al., 2010; André et al., 2014). Moreover, recently, the potential of MC to directly kill cancer cells has been highlighted (André et al., 2017). More specifically, intrathecal liposomal aracytine may have contributed to directly kill metastatic meningeal tumors cells for which inhibition of angiogenesis does not seem to be efficient since thin layers of tumors cells may not depend upon angiogenesis for survival (Peyrl et al., 2012).

## Concluding Remarks

Long term and high volume trials of metronomic chemotherapy in ATRT are not available. We offer experience in a complete and sustained clinical-radiological response to MC in a case of early-relapsing ATRT.

## Ethics Statement

This patient with a refractory disease for which no guideline at relapse are available was treated on a case to case basis. Consent was obtained from both parents and the child.

## Author Contributions

NA was in charge of the patient, directed the work and wrote the manuscript. MB wrote the manuscript. LP was the treating radiotherapist and checked the final version of the manuscript. AR was in charge of the patient and checked the final version of the manuscript. GP-G was the surgeon in charge of the patient and checked the final version of the manuscript. DF-B was the neuro-pathologist in charge of the patient and checked the final version of the manuscript.

## Conflict of Interest Statement

The authors declare that the research was conducted in the absence of any commercial or financial relationships that could be construed as a potential conflict of interest.
